# Regional characterization of energy metabolism in the brain of normal and MPTP-intoxicated mice using new markers of glucose and phosphate transport

**DOI:** 10.1186/1423-0127-17-91

**Published:** 2010-12-04

**Authors:** Emmanuelle Lagrue, Hiroyuki Abe, Madakasira Lavanya, Jawida Touhami, Sylvie Bodard, Sylvie Chalon, Jean-Luc Battini, Marc Sitbon, Pierre Castelnau

**Affiliations:** 1 UMR Inserm U 930, CNRS FRE 2448, Université François Rabelais de Tours, F-37044 Tours, France; 2 Université François Rabelais de Tours, F-37044 Tours, France; 3 Unité de Neuropédiatrie et Centre de compétence Maladies mitochondriales, Pôle Enfant, Hôpital Clocheville, CHRU de Tours, F-37044 Tours, France; 4 Institut de Génétique Moléculaire de Montpellier, CNRS UMR 5535, 1919 Route de Mende, Montpellier Cedex 5, F-34293 France; 5 Université de Montpellier 1 et 2, Place Eugène Bataillon, Montpellier, 34293 France; 6 Department of Anatomy, Teikyo University School of Medicine, 2-11-1 Kaga, Itabashi-ku, Tokyo 173-8605, JAPAN; 7 Department of Microbiology, University of Pennsylvania, Philadelphia, PA 19104-6142, USA

## Abstract

The gibbon ape leukemia virus (GALV), the amphotropic murine leukemia virus (AMLV) and the human T-cell leukemia virus (HTLV) are retroviruses that specifically bind nutrient transporters with their envelope glycoproteins (Env) when entering host cells. Here, we used tagged ligands derived from GALV, AMLV, and HTLV Env to monitor the distribution of their cognate receptors, the inorganic phosphate transporters PiT1 and PiT2, and the glucose transporter GLUT1, respectively, in basal conditions and after acute energy deficiency. For this purpose, we monitored changes in the distribution of PiT1, PiT2 and GLUT1 in the cerebellum, the frontal cortex, the corpus callosum, the striatum and the substantia nigra (SN) of C57/BL6 mice after administration of 1-methyl-4-phenyl-1,2,3,6 tetrahydropyridinium (MPTP), a mitochondrial complex I inhibitor which induces neuronal degeneration in the striato-nigral network.

The PiT1 ligand stained oligodendrocytes in the corpus callosum and showed a reticular pattern in the SN. The PiT2 ligand stained particularly the cerebellar Purkinje cells, while GLUT1 labelling was mainly observed throughout the cortex, basal ganglia and cerebellar gray matter. Interestingly, unlike GLUT1 and PiT2 distributions which did not appear to be modified by MPTP intoxication, PiT1 immunostaining seemed to be more extended in the SN. The plausible reasons for this change following acute energy stress are discussed.

These new ligands therefore constitute new metabolic markers which should help to unravel cellular adaptations to a wide variety of normal and pathologic conditions and to determine the role of specific nutrient transporters in tissue homeostasis.

## Background

Energy stress appears to be a common and early pathogenic pathway in several neurodegenerative diseases occurring in childhood or adulthood [1]. Mitochondrion, which is responsible for the adenosine triphosphate (ATP) synthesis through the mitochondrial respiratory chain (RC), plays a pivotal role when cells face energetic failure. Among all cell types, neurons show a specific vulnerability to energy stress as they display a high energy demand and are largely dependent on glucose. Importance of such mitochondrial failure has been well established in several neurodegenerative diseases in adults, including stroke, Alzheimer's disease, Parkinson's disease, Huntington's disease or amyotrophic lateral sclerosis [2]. This has been also demonstrated in several metabolic and degenerative encephalopathies in childhood, such as hypoxic-ischemic encephalopathy, iron metabolism disorders, organic acidurias or mitochondrial diseases [3-7].

In order to investigate the pathophysiological steps which occur during cerebral mitochondrial distress, we previously characterized a murine respiratory chain deficiency model using 1-methyl-4-phenyl-1,2,3,6 tetrahydropyridinium (MPTP) [8,9]. Here, we studied the regional distribution of the inorganic phosphate (Pi) and glucose transporter in the brain of normal and MPTP-intoxicated mice.

Pi and glucose represent key molecules in cellular energy metabolism. The mitochondrion membrane protein ATP synthase depends on Pi supply for ATP synthesis and Pi biodisponibility is therefore critical in cerebral homeostasis [10]. Recently, the validity of commercial antibodies directed against nutrient transporters has been questioned [11]. Thus, assessing Pi metabolism with ligands to the PiT1 and PiT2 high affinity transporters may be a more reliable approach, although PiT1 and PiT2 might exhibit different cellular functions [12]. Thus, PiT1 has been recently reported to be critical for cell proliferation, a property apparently not shared by PiT2 [13].

Several gamma and deltaretroviruses use nutrient transporters as receptors for viral entry. Viral entry is triggered after direct binding of the extracellular SU component of retroviral envelope glycoproteins (Env) to extracellular domains of the cognate transporters used as receptors [14,15]. Binding is ensured by the aminoterminal receptor binding domain (RBD) of the Env SU. Based on this phenomenon, we derived immunoadhesins from several retroviral RBD to serve as new extracellular ligands for the detection and the study of transporters of interest. We previously reported an HTLV Env RBD-based immunoadhesin (HRBD) that serves as a uniquely useful extracellular ligand of the glucose transporter 1 (GLUT1) [16,17]. Subsequently, HRBD has been largely reported to be a reliable extracellular ligand for the evaluation of GLUT1 surface distribution and intracellular trafficking in various tissues [11,18,19]. Similarly, an immunoadhesin that binds the sodium-dependent phosphate symporter PiT2 has been derived from the RBD of the amphotropic MLV (AMLV) [20,16]. Since the gibbon ape leukemia virus (GALV) uses PiT1, the other sodium-dependent phosphate symporter as receptor for viral entry, we derived a new extracellular ligand for PiT1 based on the GALV RBD [21,22].

Here, we took advantage of these transporter ligands as new metabolic markers, to monitor the distribution of GLUT1, PiT1 and PiT2 in several regions of normal and MPTP-intoxicated mice brain in order to determine whether the energy stress secondary to an acute mitochondrial dysfunction can modify the tissue distribution of theses key nutrient transporters.

## Methods

### Fusion proteins generation

We previously described HRBD, the HTLV Env RBD-derived ligand that binds the extracellular loop 6 on GLUT1 [16,15]. AmphoΔSU, an MLV Env-derived PiT2 ligand that comprises the aminoterminal 379 residues of the amphotropic murine leukemia virus Env SU fused at the carboxyterminus with rabbit IgG Fc tag(rFc) has been previously reported [20,16]. We now describe a PiT1-binding immunoadhesin generated by fusing the aminoterminal residues of the GALV (SEATO strain) Env, comprising the signal peptide, the RBD and the proline-rich region, to the rFc tag, herein, referred to as GRBD.

HRBD, AmphoΔSU and GRBD tagged ligands, and control conditioned medium were produced by transfecting 293T cells with the appropriate constructs or with the empty control vector using the calcium phosphate method [16]. After transfection, the culture medium was replaced with fresh medium without fetal bovine serum (FBS). Media containing the various soluble RBDs were harvested 2 days later and clarified by filtration (0.45 μm) to remove cell debris. The supernatants were concentrated 12-fold using an iCon concentrator 20 ml/9K spin column (Thermo Fischer Scientific, Rockford, USA). Conditioned media were frozen at -20°C until further use. Concentrated supernatants were clarified by centrifugation at 2300 g for 10 minutes at 4°C before use.

### Animals

All experiments were performed on consanguineous male C57/BL6N@Rj mice (5 weeks old, average weight: 19 ± 1 g (CERJ, Le Genest St Isle, France)) with 6 mice per group. All experiments were carried out in compliance with appropriate European Community Commission directive guidelines (86/609/EEC). Mice were kept under environmentally controlled conditions (room temperature (RT) = 23 ± 1°C, humidity = 40.3 ± 7.1%) on a 12-hour light/dark cycle with food and water *ad libitum*.

### MPTP intoxication

Mice (6 animals per group) were intoxicated with 4 administrations of MPTP (12.5 mg/kg) intraperitonealy (ip) at 1-hour intervals on a single day. MPTP (Sigma, France) was dissolved in 0.9% sodium chloride to a final concentration of 2.5 mg/ml (100 μL injection per 20 g body weight). Control mice (6 per group) were injected 4 times ip with saline. Through such regimen, MPTP induces a loss of approximately 70% of the dopaminergic neurons from the substantia nigra (SN) at day 7 after MPTP intoxication, with a combination of both necrosis and apoptosis [23]. This acute intoxication provides a validated and reliable model of energy stress which we monitor through tyrosine hydroxylase immunoreactivity and dopamine transporter density measurment as previously described [8,9].

### Immunofluorescence assays

Cryosections were generated from mice sacrificed by cervical dislocation 7 days after MPTP intoxication. Five areas of interest were studied: the cerebellum, the frontal cortex, the corpus callosum (CC), the striatum and the SN. Mouse brains were rapidly removed and frozen in isopentane (-35°C). Twenty-μm coronal sections prepared with a cryostat microtome (Reichert-Jung Cryocut CM3000 Leica Microsystems, Rueil-Malmaison, France) were collected on Super Frost Plus slides (Menzel Gläser, Braunschweig, Germany) and stored at -80°C. After fixation with 100% ethanol at room temperature, the sections were blocked with normal goat serum and endogenous biotin blocking reagent (Biotin blocking system, Dako, Via Real, CA, USA) prior to the incubation with either HRBD (ligand for GLUT1), GRBD (ligand for PiT1) or AmphoΔSU (ligand for PiT2). Several fixation protocols including 4% paraformaldehyde have been evaluated. 100% ethanol fixation was the most satisfying. Sections were incubated with the aforementioned probes for 30 minutes at 37°C. 10% FBS was added to the probes as carrier. The sections were further incubated with biotinylated anti-rabbit IgG (dilution 1/200) (Vectastain Elite kit, Vector Laboratories, Burlingame, CA, USA) for 1 h at RT, followed by incubation with Streptavidine-Alexa 488 (10 μg/ml) 30 minutes at RT, Hoechst 33342 (1 μM) (labelling for cell nucleus) and CellTrace BODIPY TR methyl ester (5 μg/ml) (labelling for intracellular membranes) (Invitrogen, Carlsbad, CA, USA) 10 minutes at RT. Negative controls were used for each reactive.

### Acquisition and restoration of the images

Brain sections were scanned with an Axio Imager Z1 upright microscope (Zeiss, Le Pecq, France). The excitation/emission filter sets specific for each of the fluorescent antibodies were as follows: <365 nm excitation filter and 420-470 nm emission filter for Hoechst (nucleus), 425-475 nm excitation filter and 485-535 nm emission filter for Alexa 488, 530-585 nm excitation filter and 615-∞ nm emission filter for CellTrace BODIPY (intracellular membranes). Image scans for each probe were acquired in seven z-series at a step-size of 3 μm with a specimen magnification of 100×. Deconvolution was performed through Huygens professional software (Scientific Volume Imaging, Hilversum, The Netherlands) with 0% background offset in order to avoid artificially decreased signals. Each plane of the individual z-series image stuck was overlaid into a three-dimensional end product. Then, two-dimensional projections were prepared by Maximum Intensity Projection on Image J software with the same display ranges for each emission in all the images. Precise measurements such as cell counts or staining quantitation were not collected for this study.

## Results

### Animals

All the animals survived during the observation period. The MPTP-induced transient weight loss observed at day 4 as expected did not cause significant differences in body weight between normal and intoxicated animals.

### Regional GLUT1, PiT1 and PiT2 distribution in the brain of normal mice

**Figure 1:**
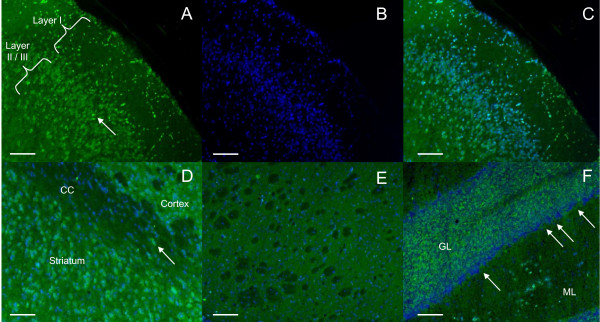
**GLUT1 immunostaining in normal mice**. Cortex immunostaining: cells within layers I to IV exhibit a cytoplasmic staining. The staining is presented as follows: A: Alexa 488 signals (green) for GLUT1. The arrow indicates an example of stained cell; B: Hoecsht signals (blue) for the nuclear counterstaining; C: Alexa 488 signals (green) and Hoechst signals (blue) are merged; D: Corpus callosum (CC) staining: a few stained oligodendrocytes are seen (arrow). (Alexa 488 signal and Hoechst signals merged); E: Striatum staining: GLUT1 staining appears homogeneous and weak with few cellular bodies stained. The white-matter tracts are not labeled for GLUT1. (Alexa 488 signal and Hoechst signals merged); F: Cerebellum staining: The granular layer (GL) and the molecular layer (ML) are irregularly labelled for GLUT1, whereas the molecular layer is homogeneously labelled for PiT1 and PiT2. (Alexa 488 signal and Hoechst signals merged). Scale bar: 100 μm.

**Figure 2:**
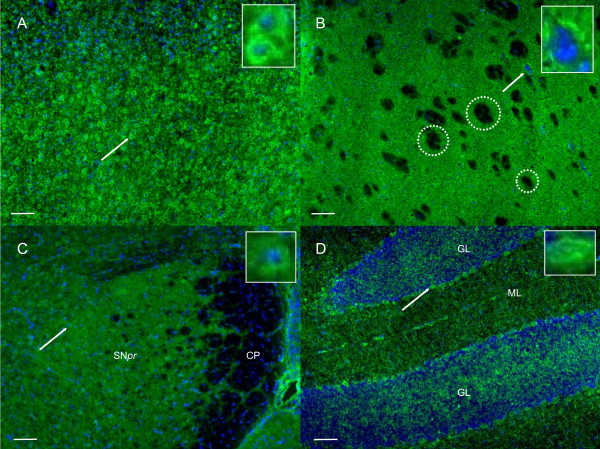
**PiT2 immunostaining in normal mice**. A: PiT2 immunostaining in the cortex of a normal mouse. In this representative image, the staining is detected in all cortical layers, with a "rosette like" aspect. The arrow indicates a characteristic stained neuron displayed in the enlarged inset (magnification x300). B: PiT2 immunostaining in the striatum of a normal mouse. Some PiT2-stained cells carry a "rosette like" pattern similar to that observed in the cortex (arrow and enlarged inset, magnification x300). Noteworthy, the white matter tracts are not stained (shown within dotted circles). C: PiT2 immunostaining in the substantia nigra (SN) of a normal mouse. PiT2 staining pattern in SN is comparable to the patterns observed in the cortex and the striatum with a "rosette like" aspect. The cerebral peduncle (white matter) does not show any PiT2 staining. The arrow points at a characteristic stained nigral cell as shown in the inset (magnification x300). D: PiT2 immunostaining in the cerebellum of a normal mouse. Purkinje cells are labelled with the PiT2 specific probe (arrow). Alexa 488 signals for PiT2 (green) and Hoechst signals for the nuclear counterstaining (blue) are merged. CP: cerebral peduncle, SNpr: substantia nigra *pars reticulata*, ML: molecular layer, GL: granular layer. Scale bar: 100 μm.

**Figure 3:**
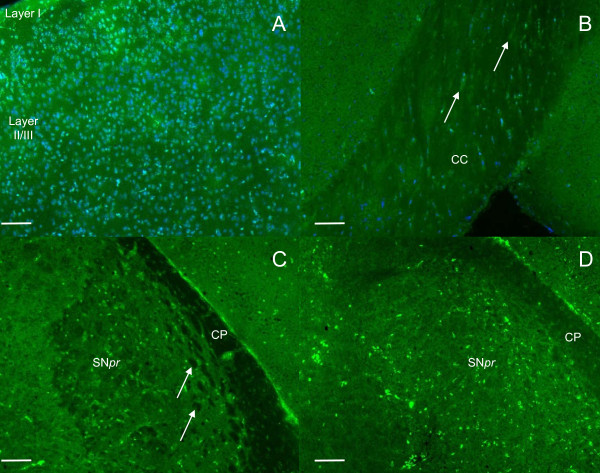
**PiT1 immunostaining in normal and MPTP-intoxicated mice**. A: PiT1 staining in the cortex of control mice; stained neurons are mostly detected in layer II/III. These neurons are medium-sized with homogeneous cytoplasmic staining. B: PiT1 immunostaining in the corpus callosum (CC) of normal mice: PiT1 labelling exhibits a linear pattern with few stained cells following the myelinated fiber bundles corresponding to oligodendrocytes (arrows). C: PiT1 immunostaining in the SN of normal mice with a reticular pattern due to a relative sparing of white-matter (arrows). D: PiT1 immunolabelling in MPTP intoxicated mice where an apparent extension of staining can be seen in the white-matter bundles in the substantia nigra *pars reticulata *(SNpr) and in the cerebral peduncle (CP). The staining is presented as follows: A to D, staining with Alexa 488 (green, PiT1 ligand) and A and B, signals are merged with Hoechst (blue, counterstaining for nuclei). Scale bar: 100 μm.

Cortex staining: GLUT1 staining was heterogeneous from layer I to IV: layer I exhibited a low cellular density and all the neuronal cells in this layer were apparently stained. Layer II/III displayed a higher cellular density compared to layer I with general cytoplasm staining. However, the staining intensity was different from one cell to another. Representative microphotographs of GLUT1 immunostaining in the cortex of normal mice are shown in Figure 1A-C. PiT2 labelling gave a different pattern: the staining was detected in layer I to IV and was exclusively peripheral with a "rosette like" aspect (Figure 2A). As for PiT1, staining in the cortex varied from layer I to IV with stained neurons predominantly detected in layer II/III. These neurons were medium-sized with a homogeneous cytoplasmic staining (Figure 3A).

Corpus callosum staining: A few GLUT1-labelled cells were seen (Figure 1D) with a weak staining compared visually to the cortex and striatum. No PiT2 staining was observed (not shown). Perivascular cells were markedly labelled with the GLUT1 and PiT2 ligands. PiT1 staining exhibited a linear pattern with few stained cells following the myelinated fiber bundles corresponding to oligodendrocytes (Figure 3B).

Basal ganglia staining: In the striatum, GLUT1 labelling appeared rather weak and homogeneously diffuse (Figure 1E). PiT1 labelling was also weak and detected only in a few cellular bodies (4-5 cells in each striatum) (data not shown). PiT2 staining was distinct, with a "rosette like" pattern similar to that observed in the cortex in addition to the diffuse staining throughout the striatum (Figure 2B). Noteworthy, the white matter tracts were not stained with any of the three markers. In the Substantia Nigra: no distinct binding of the GLUT1 ligand was detected, with the structure rather presenting a diffuse staining (data not shown). PiT1, on the other hand, showed a reticular pattern with several stained cellular bodies (Figure 3C). PiT2 staining was comparable to the ones observed in the cortex and the striatum with a "rosette like" aspect (Figure 2C). As observed within the CC, the cerebral peduncle, corresponding to white matter, did not show any GLUT1 or PiT2 staining, whereas several oligodendrocytes were detected by PiT1 staining.

Cerebellum staining: the granular layer was irregularly labelled with all three probes, whereas the molecular layer was homogeneously labelled for PiT1 and PiT2 and irregularly labelled for GLUT1. The Purkinje cells were irregularly labelled for GLUT1 (Figure 1F), PiT1 and PiT2 (Figure 2D).

### Regional GLUT1, PiT1 and PiT2 distribution in the brain of MPTP-intoxicated mice

No noticeable change was observed in PiT1, PiT2 and GLUT1 distribution in the cortex, the CC, the striatum and the cerebellum after MPTP administration (data not shown).

In the SN *pars reticulata*, GLUT1 and PiT2 staining were unchanged in comparison to normal mice brain. Conversely, the PiT1 distribution pattern in the SN was modified after MPTP administration: The cell density and staining did not appear to be altered but the reticular pattern, observed in normal mice brain, was not anymore detected due to a labelling of the white-matter fiber tracts apparently recruited and newly stained, including the cerebral peduncle (Figure 3D).

## Discussion

Here, we took advantage of new retroviral Env-derived markers for nutrient transporters to detect directly and for the first time the regional distribution of glucose and phosphate transporters in mouse brain during energy stress. MPTP was used to induce such aggression through an acute respiratory chain deficiency.

### Regional GLUT1 distribution in basal conditions

With HRBD, the GLUT1 ligand, we observed a staining of GLUT1 in the corpus callosum and the basal ganglia apparently weaker than in the cerebellum and in the cortex.

These results were reproducible in all animals and are in accordance with the literature: the detection of GLUT1 by immunoblotting performed in rats has previously shown that GLUT1 is expressed in all brain regions but in less abundance in the striatum, the thalamus and the brainstem [24]. In mice, only blood vessels were found to be immunostained using an antibody raised against the C-terminal part of the protein [25,26]. Cell surface antibodies directed against metabolite transporters are rare because of high inter-species homology and low immunogenicity of the external loops. Our metabolic markers, all interact with extracellular determinants of the multimembrane-spanning transporter molecules. It must be specified that our markers are independent from N-glycosylation variations and that our GLUT1 ligand, HRBD, does not cross-react with GLUT3 or other GLUT isoforms [16,15]. However, we cannot formerly exclude that a lack of labeling may not be due to the absence of cell surface expression of the transporter but merely to a cell surface environment than hinders ligand binding. Thus, it has previously been shown that a general inhibition of cell glycosylation by tunicamycin allowed receptor recognition and infection driven by an MLV envelope [27]. Whether, a lack of staining may come from an absence of receptor/transporter or an altered accessibility remains to be determined. In any case, lack of staining reflects major changes in the transporter environment and in the case of GLUT1, such changes have been shown to have a major impact on GLUT1 transporter functions [19].

### Regional PiT distribution in basal conditions

To our knowledge, this is the first time that the regional distribution of PiT1 and PiT2 were monitored in normal mouse brain through immunofluorescence methods. We observed that, although both PiT1 and PiT2 have been described as inorganic phosphate transporters, they show distinctive distribution patterns. Cells appearing to be oligodendrocytes were labelled with PiT1 but not PiT2. In the SN, PiT1 showed various stained cellular bodies with a reticular pattern suggesting a sparing of white-matter bundles, whereas the PiT2 staining pattern was comparable to the one observed in the cortex and the striatum with a "rosette like" aspect. Hence, our results represent a regional study which needs to be further explored at the cellular level. The differential distribution pattern for PiT1 and PiT2 might reflect a difference in cellular functions between PiT1 and PiT2. This issue has been recently highlighted when PiT1, unlike PiT2, was reported to be critical for cell proliferation, independently of their common phosphate transport activity [13]. Recently, Festing et al generated the first conditional and null PiT1 allele mouse and observed that the hemizygous PiT1 knock-out is lethal. Since the expression of PiT2 gene was not modulated in the affected tissues in compensatory ways, these authors conclude that PiT1 carries an essential and non redundant role in embryonic development [28]. Altogether, these data might suggest various regulations of the different inorganic phosphate transporters which are likely to indicate unique functional roles for each one.

### Regional GLUT1 distribution after energy stress

We subsequently studied the changes of PiT1, PiT2 and GLUT1 distribution after MPTP intoxication. As MPTP specifically induces a basal ganglia degeneration [23,9], we focused on GLUT1 changes in these structures. We observed that under a basal energy state, there was a homogeneous GLUT1 distribution in the striatum and the SN that remained identical after MPTP intoxication. However, GLUT1 is known to be down-regulated by mitochondrial inhibitors in some animal cultured cell lines [29]. Such an apparent discrepancy may be related to the sensitivity of our technique which may not allow the study of limited variations in discrete areas such as the SN *pars compacta*. Alternatively, it is also plausible that in order to change GLUT1 transporter expression in the SN, the energy stress should be more prolonged or pronounced than in the acute intoxication which we tested. To evaluate the consequences of a prolonged energy insult, a chronic MPTP regimen should be used [23].

### Regional PiT distribution after energy stress

We observed that PiT1 tissue distribution was modified and appeared to be more extended in the SN after MPTP intoxication. Several hypotheses may be raised to explain the exact significance of such observation:

The fact that we observed PiT1 redistribution in all the intoxicated animals and in no other area we monitored except the SN, where MPTP toxicity specifically takes place, supported the validity and specificity of our observation. Also, the fact that the white-matter bundles seemed to be recruited specifically at two different sites also strongly argued in favor of specific labelling that reflects *de novo *expression of this transporter in precisely delineated structures, namely the SN and the cerebral peduncles, where PiT1 normally appears to be quiescent. Phosphate homeostasis is necessary for ATP production through the mitochondrial RC. Interestingly, the enzyme responsible for ATP synthesis, ATP synthase (or complex V), is associated with the phosphate carrier (PIC), which transport Pi, and the adenine dinucleotide carrier (ANC), which transport ADP, in a large protein complex called ATP synthasome [30-32]. The ATP synthase then combines ADP and Pi to form ATP. Therefore, an increase in the cytosolic Pi content is likely to promote ATP synthesis and, thereby, counteract energy deficiency and a subsequent cellular degeneration. The apparent extension of PiT1 expression in the SN could translate a neuroprotective adaptation to increase ATP synthesis where MPTP deprives neurons from their energy supplies. Although difficult to perform in mice brain, a specific measurement of the complex V activity in the SN would provide important information to support such hypothesis. Moreover, since PiT1 has been shown to be critical for cell proliferation [33], an upregulation of PiT1 might indicate an attempt to promote cell survival and rescue, especially in the white matter where a compensatory sprouting from the dopaminergic nigral projections toward the striatum, has been largely described in immediate response to MPTP toxicity [23,8].

Conversely, one could postulate that such modification in PiT1 pattern of distribution participates to the sequence of lesions in the SN and rather traduces MPTP toxicity. Indeed, PIC is a key component of the mitochondrial permeability transition pore [34]. The apparent extension of PiT1 distribution could generate detrimental changes in PIC regulation and, thereby, in the ATP synthasome homeostasis. An alteration in the formation of this huge protein complex could release PIC molecules and, subsequently, enhance mitochondrial transition pore opening which involvement in MPTP toxicity has been shown to participate to a combination of necrotic and apoptotic cell death [23]. Consistently, a direct effect of MPTP on PiT1 expression cannot be also excluded at present.

Unlike for PiT1, the PiT2 distribution was not modified after MPTP intoxication. This would be consistent with the fact that a differential regulation of Pi transporters takes place in the brain, in basal but also pathologic conditions [13].

A natural neuroprotective reaction occurring in the SN after MPTP intoxication is also conceivable, but this would need to be confirmed by studies at the cellular level including kinetic studies to further determine the regulation of the inorganic phosphate transporters in the brain.

In conclusion, our data suggest that these new metabolic markers can be used to improve our understanding of the metabolism in the brain, as well as in others organs such as the heart, the liver or kidneys. In addition, these new ligands could help a better understanding of the role of their cognate transporters. It is also important to note that these transporters are multifunctional proteins: Hence, GLUT1 also transports the oxidized form of ascorbic acid, dehydroascorbic acid (DHA), in mammals which are unable to synthesize vitamin C [19,35]. PiT, alternatively, can transport zinc in the bacteria E. Coli [36]. Interestingly, vitamin C and zinc support major pathophysiological pathways: vitamin C is an endogenous antioxidant [37] and zinc is the cofactor of more than 300 enzymes. High levels of labile zinc accumulate in degenerating neurons after brain injury, such as ischemic stroke, trauma, seizure and hypoglycaemia [38]. Excessive levels of free ionic zinc can initiate DNA damage and the subsequent activation of poly(ADP-ribose) polymerase 1 (PARP-1), which in turn leads to NAD+ and ATP depletion when DNA damage is extensive [39]. Zinc also modulates hippocampic neurogenesis [40]. Since these nutrient transporters are involved in various pathways of neurodegeneration/neurogenesis, their study might, therefore, provide additional insights in the natural mechanisms of cellular defence and lead, thereby, to the conception of new neuroprotection strategies.

## Competing interests

The authors declare that they have no competing interests.

## Authors' contributions

EL and HA: carried out the immunofluorescence assays and drafted the manuscript; JLB and MS: conceived the envelope-derived tagged ligands while; JLB, HA, ML and JT: generated, optimized and produced these ligands; SB: participated to the animal experiments; SC: participated to the initiation of the study; MS and PC: conceived the study, organized the experimental schedule and conducted the manuscript writing. All authors have read and approved the final version of the manuscript.
